# Rheumatoid arthritis and gastroesophageal reflux disease: a bidirectional and multivariable two-sample Mendelian randomization study

**DOI:** 10.3389/fgene.2023.1280378

**Published:** 2023-12-13

**Authors:** Haifan Wang, Zhihao Chen, Xiaoqian Dang, Haoyu Wang

**Affiliations:** Department of Orthopaedics, The Second Affiliated Hospital of Xi’an Jiaotong University, Xi’an, Shaanxi, China

**Keywords:** rheumatoid arthritis, gastroesophageal reflux disease, Mendelian randomization, education, BMI

## Abstract

**Aims/hypothesis:** The association between gastroesophageal reflux disease (GERD) and rheumatoid arthritis (RA) has been reported by many observational studies in the Asian population. This study aimed to examine the bidirectional causal effects between GERD and RA by two-sample Mendelian randomization (MR) analyses using genetic evidence.

**Methods:** Two-sample Mendelian randomization analyses were performed to determine the causal effect of GERD (129,080 cases vs. 602,604 control participants) on RA (6,236 cases vs. 147,221 control participants) and RA on GERD, respectively. The inverse-variance weighted (IVW) method was used as the primary analysis. Weighted median and MR-Egger regression were taken as supplementary analyses. Cochran’s Q test evaluated the heterogeneity. Horizontal pleiotropy was detected by estimating the intercept term of MR-Egger regression. Furthermore, multivariable MR analyses were performed to exclude the influence of confounding factors, including the years of schooling, BMI, and time spent watching television, between GERD and RA.

**Result:** Both univariate MR (UVMR) and multivariable MR (MVMR) provided valid evidence that RA was causally and positively influenced by GERD (UVMR: OR = 1.49, 95% CI = 1.25–1.76, *p* = 6.18*10^−6^; MVMR: OR = 1.69, 95% CI = 1.24–2.31, *p* = 8.62*10^−4^), whereas GERD was not influenced by RA (UVMR: OR = 1.03, 95% CI = 1.00–1.06, *p* = 0.042; MVMR: OR = 1.04, 95% CI = 1.00–1.07, *p* = 0.0271).

**Conclusion:** Our comprehensive bidirectional MR analysis found that for the European population, GERD can induce the occurrence of RA (OR = 1.69, *p* < 0.00125), whereas RA only has no significant influence on GERD. In particular, patients with GERD are suffering a 69% increased risk of RA occurrence, which means GERD is a substantial risk factor for RA.

## Introduction

Rheumatoid arthritis (RA) mainly affects the joints, with extra-articular tissues being involved (Smolen et al., 2016). The incidence of RA is estimated to be approximately 0.1–0.5 per 1,000 person/year, which varies according to the ethnic group (Tobón et al., 2010). The extra-articular manifestations revealed the existence of systemic inflammation in RA (Smolen et al., 2022). Apart from inherited susceptibility, low socioeconomic status, periodontal diseases, and microbiome are also the risk factors of RA (Millar et al., 2013; Scher et al., 2016; Li et al., 2017). Those risk factors may influence systemic inflammation to induce RA.

Gastroesophageal reflux disease (GERD) is a common disease that results from the reverse flow of stomach acid into the esophagus (Vakil et al., 2006). Nearly one-fifth of North American people are suffering from GERD, which is nearly four times more prevalent than in Asian populations. This disease causes distressing symptoms, such as heartburn, inappetence, nausea, and susceptibility to pharyngitis, and some other diseases (Punjabi et al., 2015). Mechanically, GERD is primarily caused by abnormal physiology and anatomy changes in the stomach and esophagus. These changes include an increased pressure gradient between the abdomen and thorax, dysmotility of the esophagus, hiatus musculature, and/or the stomach. As a result, the normal reflux barrier of the LES breaks down (Mikami and Murayama, 2015).

In addition to anatomical and physiological factors, inflammation occurring in the stomach and esophagus plays a crucial role in the development of GERD (Punjabi et al., 2015; Souza et al., 2017; Surdea-Blaga et al., 2019). Consistently, an association between GERD and systemic inflammatory diseases has been reported (Chen, 2015; Linz et al., 2017), particularly for RA (Cryer et al., 2011; Nampei et al., 2013; Lin et al., 2017). In detail, some observational research found a higher incidence of GERD in patients with RA (Cryer et al., 2011; Lin et al., 2017). In Japan, more than 2-fold higher incidence of GERD in patients with RA than that in normal people has been reported (Lin et al., 2017). Meanwhile, it has also been reported that patients with GERD exhibit a nearly 3-fold higher risk of RA than those in the control group in the Taiwan population (Lin et al., 2017). The bidirectional association between GERD and RA has been identified. One cohort study performed on the Asian population reported an HR of 1.49 for RA in patients with GERD and 1.46 for GERD in patients with RA (Kim et al., 2021).

Mendelian randomization (MR) analysis utilizes single-nucleotide polymorphisms (SNPs) to find the causality between risk factors and outcomes (Lawlor et al., 2008). The superiority of MR lies in the fact that SNPs are determined before the intervention of the environment. Thus, they can be used as proxies for phenotypes and diseases (Davey Smith and Hemani, 2014). Due to its reduced susceptibility to reverse causation and confounding, MR conclusions are considered more reliable than those of conventional observational studies (Davey Smith and Hemani, 2014).

The prerequisites for MR are based on three assumptions: first, IVs should be strongly associated with exposure; second, IVs should influence the outcome only through the exposure (no horizontal pleiotropy); and third, IVs should not be associated with confounders. The two-sample MR tests the causality based on the GWAS data risk factors, and outcomes are measured in their respective samples (Boef et al., 2015). After searching in reference databases, we found that MR has not been applied to explore the causal effects between GERD and RA.

However, the relationship between GERD and RA has not been observed in European population and evaluated using the MR method. In this study, univariate and multivariable bidirectional two-sample MR analyses were performed to test the reciprocal causal relationship between GERD and RA.

## Methods

### Study design

**FIGURE 1:**
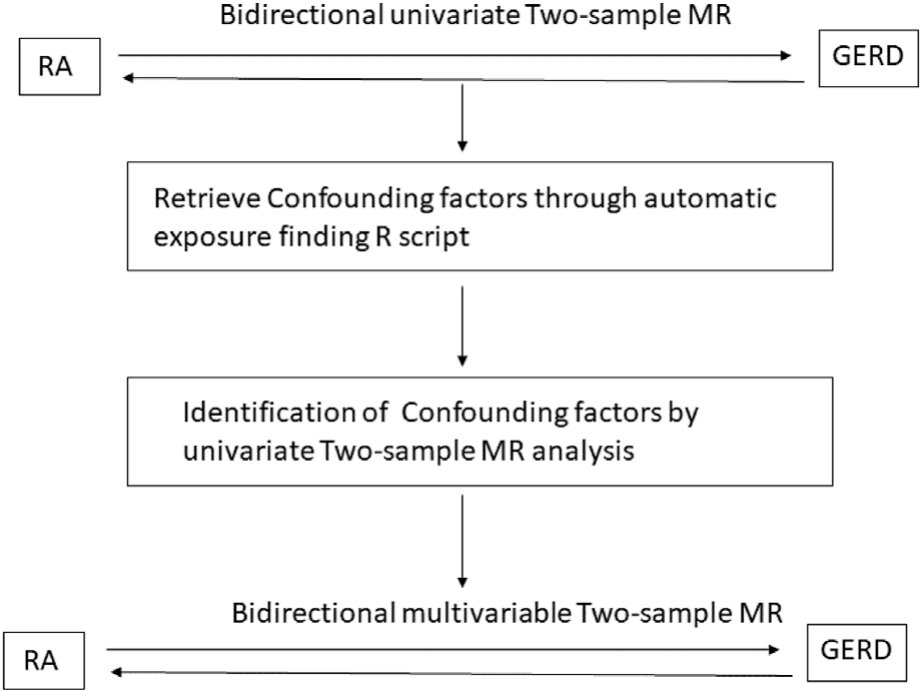
Workflow of this study design is shown in the sketch map.

As shown in Figure 1, first, the univariate bidirectional MR analysis of the causal relationship between GERD and RA was performed. When exposure was set as GERD, RA was considered to be the outcome. When exposure was set as RA, GERD was considered to be the outcome. Furthermore, confounding factors between GERD and RA were retrieved. In detail, the mutual potential exposure relationship between GERD and RA was found by batching the processing TwoSample-MR R script. Then, three confounding factors were included through the automatic exposure finding R script. Finally, three confounding factors are selected: years of schooling, BMI, and time spent watching television.

For UVMR, the exposure SNPs (*p* < 5 × 10^−8^, r2 < 0.001, F > 10 for GERD and *p* < 1 × 10^−5^, r2<0.001, F > 10 for RA) were selected as instrumental variables. Furthermore, sensitivity and pleiotropy analyses were performed to ensure the robustness of the results. For multivariable MR (MVMR), the selection threshold of the mv_extract_exposures function in the TwoSample-MR package was set as default.

### Data source

All data involved are publicly available. The RA GWAS data including 153,457 European individuals (6,236 cases vs. 147,221 control participants) retrieved from the IEU database (id: finn-b-M13_RHEUMA) were originally derived from the Finn-gen Consortium [Trait: Duration of vigorous activity – IEU OpenGWAS project. 2021. https://gwas.mrcieu.ac.uk/datasets/ukb-b-13932/ (1 December 2020, date last accessed), n.d.]. The GERD GWAS data including 129,080 cases and 602,604 control of European individuals were also retrieved from the IEU database (id: ebi-a-GCST90000514) originally derived from the EBI Consortium. The F-statistic of SNPs was calculated by the formula to select strong IVs (F = R2× (N−2)/(1−R2) (Chen et al., 2022). Then, we selected SNPs with an F-statistic larger than 10 to prevent potential weak instrument bias. Three confounding factors from GWAS data were retrieved from the IEU website: body mass index (id:ieu-a-94), years of schooling (id:ieu-a-1239), and time spent watching TV (id:ukb-b-5192).

### Instrumental variable selection

SNPs were filtrated by the TwoSampleMR packages of R software. Genome-wide SNPs that are closely associated with education duration were acquired by the extract_instruments function (thresholds were set as *p* < 5 × 10^−8^, r2 < 0.001, window size = 10000 kb for GERA, and *p* < 1 × 10^−5^, r2<0.001, window size = 10000 kb for RA).

### Statistical analyses

The inverse variance-weighted (IVW) method was considered as the main MR analysis to initially estimate the causal relationship of education duration on joint pain and sciatica with lumbago. The IVW method’s robustness depends on IV’s pleiotropy. Furthermore, another two MR analyses, namely, weighted median (WM) and MR-Egger, were selected as supplementary analyses to detect causalities. The WM method can estimate unbiased causality, with more than 50% of the weight coming from valid instrumental variables (Bowden et al., 2016), whereas MR-Egger estimates consistently account for pleiotropy when all IVs are invalid with the lowest power (Bowden et al., 2015). Our MR estimates of the risk of GERD or RA were presented as follows: odds ratio (OR), 95% confidence interval [CI]. A two-sided value of *p* < 0.05 is considered statistically significant for UVMR and *p* < 0.0125 for MVMR (four exposures).

### Sensitivity analysis

Cochran’s Q test, MR-Egger intercept tests, leave-one-out (LOO) analyses, and funnel plots were performed to examine the presence of pleiotropy in the results. In particular, Cochran’s Q test was applied to evaluate heterogeneity, which was detected if the *p*-value was less than 0.05. The horizontal pleiotropy of both UVMR and MVMR was appraised by estimating the intercept term derived from MR-Egger regression. The LOO analysis was performed to detect any pleiotropy driven by a single SNP. All these MR analyses were performed using the TwoSampleMR package in R.

## Results

### Univariate MR result of GERD on RA

**FIGURE 2:**
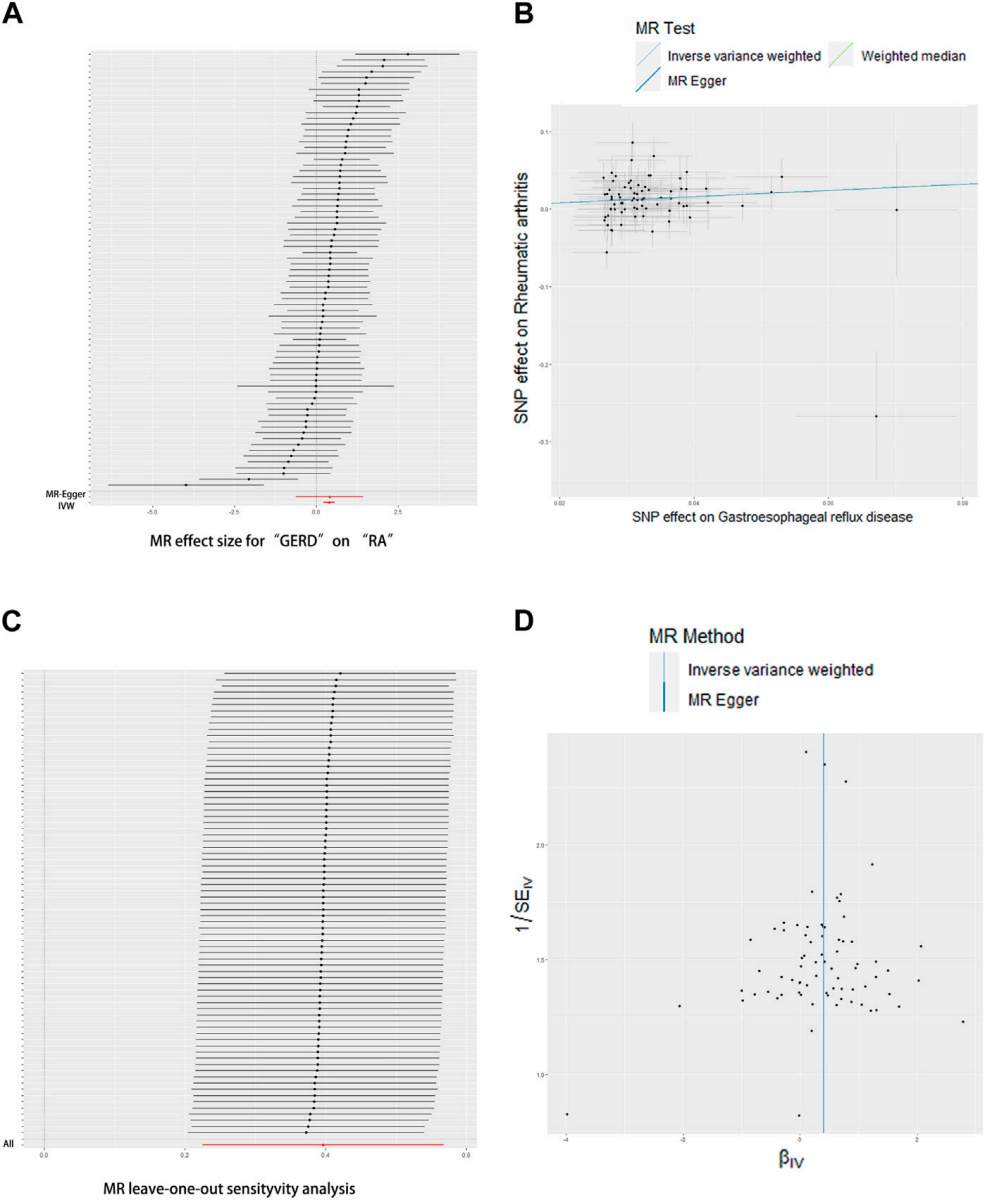
Effects of GERD on RA. **(A)** Forrest plot, **(B)** scatterplot, **(C)** leave-one-out plot, and **(D)** funnel plot.

**FIGURE 3:**
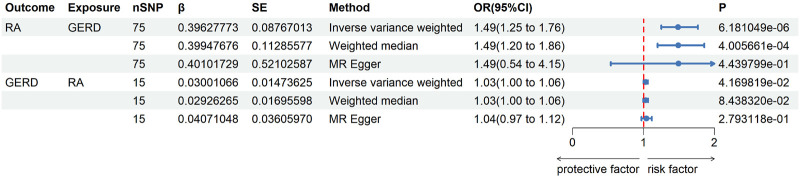
Univariate two-sample MR analysis between GERD and RA.

The UVMR results of education duration on joint pain are shown in Figures 2, 3. A total of 75 SNPs were selected as instrumental variables. Given the IVW method, RA was casually influenced by GERD (OR = 1.49, 95% confidence interval [CI] = 1.25–1.76, *p* = 6.18*10^−6^), suggesting that patients with GERD are suffering a 49% increased risk of RA occurrence. This result was consistent with the weighted median (OR = 1.49, 95% CI = 1.20–1.86, *p* = 4.00*10^−4^). Heterogeneity was not found in the effect of GERD on RA using Cochran’s Q test (*p* = 0.794), and directional pleiotropy is not existent in the SNPs associated with GERD *via* MR-Egger regression (intercept = −0.023 *p* = 0.3531). The result of leave-one-out analyses shows that the global effect of GERD on RA was not dependent on any single IV. The symmetrical funnel plots suggested that there was no significant bias in SNP selection.

### Univariate MR result of RA on GERD

The UVMR results of RA on GERD are shown in Figure 3. Only 15 SNPs were available as instrumental variables. Even the IVW method results show a *p*-value that is slightly lower than 0.05; effect value β is very small (0.03–0.04) in all three methods. For the IVW method, the GERD was slightly casually influenced by RA (OR = 1.03, 95% [CI] = 1.00–1.06, *p* = 0.0844), suggesting that both weighted median (OR = 1.03, 95% CI = 1.00–1.06, *p* = 0.2793) and MR Egger (OR = 1.04, 95% CI = 0.97–1.12, *p* = 0.2793) analyses did not support the above results. Heterogeneity was not found in the effect of RA on GERD using Cochran’s Q test (*p* = 0.0.52), and directional pleiotropy is non-existent in SNPs associated with RA *via* MR-Egger regression (intercept = −0.0016, *p* = 0.7486). In other words, there is no strong enough evidence to support that RA can induce the occurrence of GERD.

### Confounding factors and their effects

**FIGURE 4:**
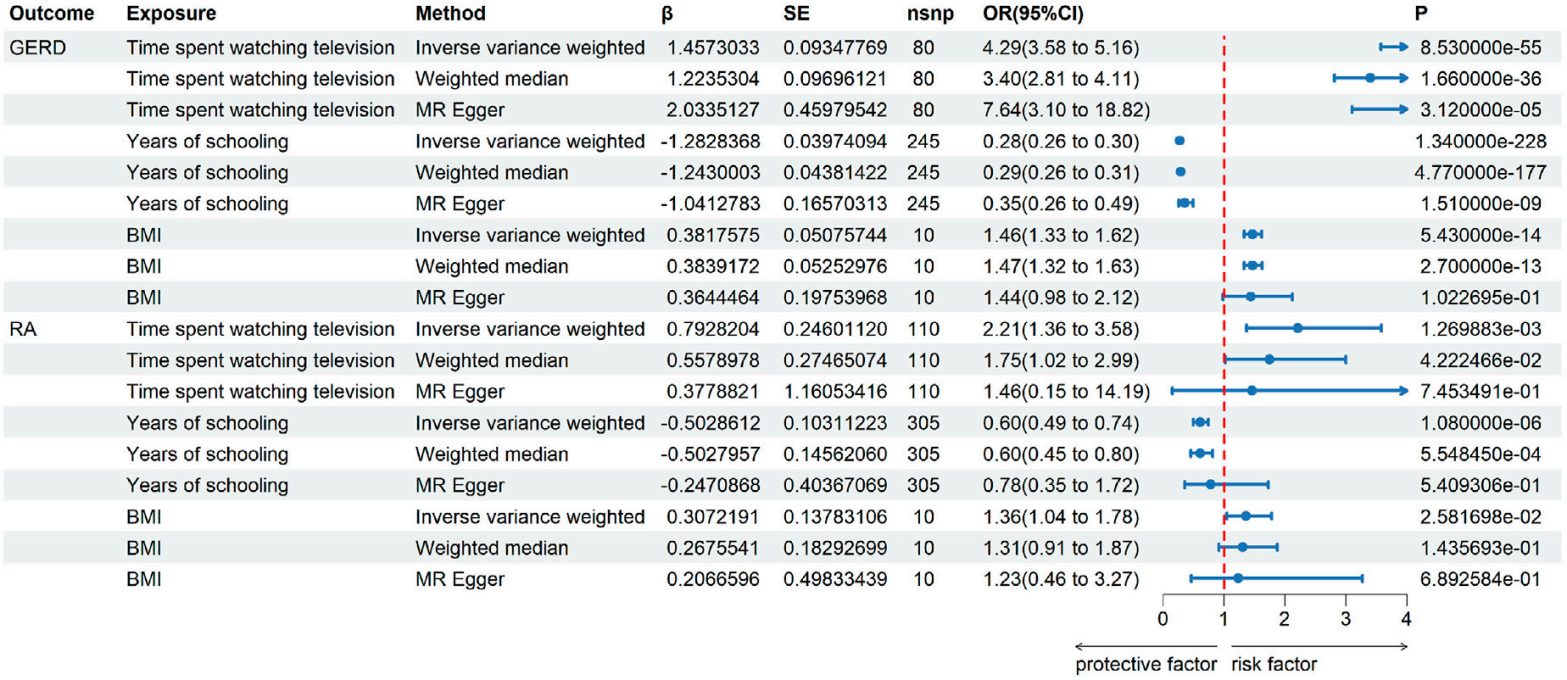
Univariate two-sample MR analysis of confounding factors’ effects on RA and GERD.

For further MVMR analysis, confounding factors are retrieved through automatic exposure finding the R script, and the two-sample MR results of those confounding factors are shown in Figure 4. It implies that all the three factors that were selected are potential confounding factors with positive two-sample UVMR analysis results.

### Multivariable MR analysis between GERD and RA

**FIGURE 5:**
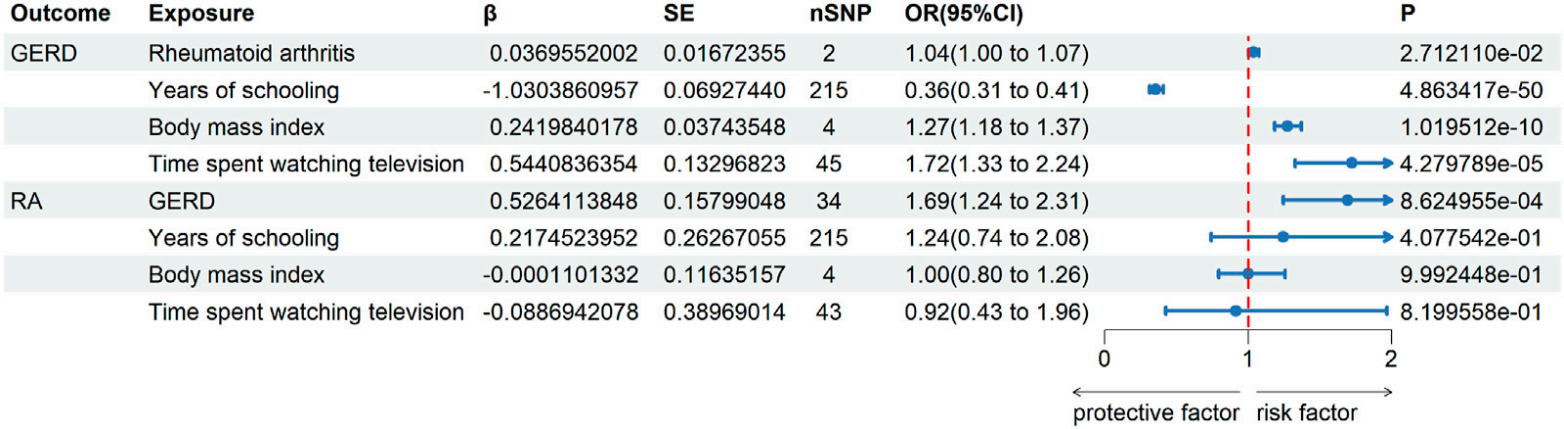
Result of MVMR analysis between GERD and RA.

The bidirectional MVMR results are shown in Figure 5. After removing the influence of confounding factors, the causal relationship of GERD on RA still exists (OR = 1.69, 95% CI = 1.24–2.31, *p* < 0.0125). Consistent with UVMR, RA only has slight effects on GERD (OR = 1.04, 95% CI = 1.00–1.07, *p* = 0.0271). It is worth mentioning that the years of schooling is an effective protective factor for GERD (OR = 0.36, 95% CI = 0.31–0.41, *p* < 0.0125), but BMI (OR = 1.27, 95% CI = 1.18–1.37, *p* < 0.0125) and time spent watching TV (OR = 1.72, 95% CI = 1.33–2.24, *p* < 0.0125) are risk factors for GERD. Directional pleiotropy was not detected in both MVMR of GERD (intercept = −0.002, *p* = 0.668) on RA and RA on GERD (intercept = 0.001, *p* = 0.130).

## Discussion

In order to examine the potential reciprocal causal relationship between GERD and RA, bidirectional two-sample Mendelian randomization analyses were conducted. The results suggested that GERD can induce the occurrence of RA, whereas RA has no significant impact on GERD. In particular, individuals with GERD are at a 69% higher risk of developing RA, highlighting GERD as a significant risk factor for this condition. In addition, the impact of three confounding variables, namely, educational attainment, BMI, and duration of television viewing, on both GERD and RA has also been identified.

The association between GERD and RA has been reported by many observational studies, as mentioned in the Introduction part. Those observational studies reported bidirectional causal relationships between GERD and RA. However, our bidirectional two-sample Mendelian randomization analyses only identified the causal effects of GERD on RA and not *vice versa*.

There are some potential common risk factors for both GERD and RA. Inclusion bias may arise when the observational studies fail to exclude those mutual factors (Thombs et al., 2011). In order to incorporate confounding factors effectively, this study conducted a preliminary two-sample MR analysis with GERD and RA as separate outcomes. This step aimed to screen for common risk factors before proceeding to the MVMR analysis. Considering the evidence level of observational studies, MR analysis, and the superiority of MVMR (Zhou et al., 2023), results of this study are influenced by fewer confounding factors. When cross-sectional and cohort studies found the reciprocal association between GERD and RA, those known and unknown confounding factors are often not excluded.

For instance, a cohort study identifying the reciprocal association between GERD and RA only matched factors, including age, group, sex, income group, income group, and region of residence (Kim et al., 2021). However, smoking, BMI, diabetes, *etc*., are not taken into account, which can result in selection bias (Punjabi et al., 2015). The control group selected in those studies often exhibits fewer risk factors of GERD that are not caused by RA. Coincidentally, the HR (1.49) of RA in patients with GERD calculated by one cohort study was approximate to our UVMR result, but the HR (1.46) of GERD in patients with RA is not found in our study (Kim et al., 2021). Recently, a meta-analysis research based on cohort studies has also reported an OR of 1.98 for GERD in patients with RA (Thongpiya et al., 2023). It is important to note that the observational studies utilized in the meta-analysis were predominantly conducted on the Asian population, whereas our MR analysis is based on the European population. In addition, the prevalence of GERD in North America is nearly 4-fold in the Asian population (Chen, 2015). This difference in population may also account for the difference between our study and former observational research.

On the other hand, patients with RA tend to take more NSAIDS than their control groups because of the symptom of RA (Burmester and Pope, 2017; Aletaha and Smolen, 2018; Ben Mrid et al., 2022). Meanwhile, the common adverse reaction of NSAIDS is gastrointestinal, which may induce GERD (Altman et al., 2015; García-Rayado et al., 2018; Bindu et al., 2020). As a result, it is natural to hypothesize about the causal effects of RA on GERD. However, our results found no significant effect of RA on GERD, unlike the obvious effect reported by previous observational studies.

Similarly, for the causal effect of GERD on RA, ORs 1.49 and 1.69 calculated in this study were smaller than the HRs reported by other observational studies. For instance, a nearly 3-fold risk of RA susceptibility in patients with GERD than their control groups was reported by a nested case–control study in the Asian population (Lin et al., 2017). Even this study has considered many known confounding factors, including hypertension, diabetes, smoking, hyperlipidemia, obesity, stroke, and coronary heart disease; when those factors (*p* > 0.1 between patients with GERD and their controls) combined, inclusion bias may also derive.

Mechanistically speaking, the physiological and anatomical changes, which may not appear directly associated with RA, have the potential to increase the risk of developing RA due to the persistent inflammation and immune dysregulation observed in GERD. However, further basic and clinical studies are required to substantiate these assumptions. In addition, the MVMR analysis also revealed that years of schooling is an effective protective factor for GERD (OR = 0.36, 95% CI = 0.31–0.41, *p* < 0.0125). Conversely, BMI (OR = 1.27, 95% CI = 1.18–1.37, *p* < 0.0125) and time spent watching TV (OR = 1.72, 95% CI = 1.33–2.24, *p* < 0.0125) are identified as risk factors for GERD. The education duration has been consistently reported as a protective factor for many diseases and phenotypes, including low back pain, RA, and lifestyle (Jiang et al., 2015; Saper et al., 2017; Davies et al., 2019; Kari et al., 2020; Zhao et al., 2022), while excessive high BMI and longer time spent watching TV have been found to be detrimental to health in many studies (Antonopoulos et al., 2016; Caballero, 2019; Raichlen et al., 2022; Sun et al., 2023). Although the effects of BMI and education on RA are detected in the search of confounding factors by UVMR searching, the MVMR analysis did not find significant effects of them on RA. This finding may support the notion that the previously reported effects of BMI and education on RA were also generated by bias factors.

The strength of this study is that the confounding factors are included in the identification of bidirectional causal relationships between GERD and RA through MR analysis. To the best of our knowledge, it is the first time to investigate their association using genetic evidence, despite previous observational studies reporting a bidirectional association between RA and GERD (Miura et al., 2014).

However, there are several limitations to this study. The GWAS data used in this study were derived from the European population, whereas most previous observational studies on the topic are based on the Asian population. Whether our findings are generalizable to non-European populations still needs to be confirmed. In addition, even though the sample of GWAS data used in this study was large, more extensive and new GWAS data may produce different conclusions.

## Conclusion

In conclusion, our bidirectional MR analysis found that for the European population, GERD can induce the occurrence of RA (OR = 1.69, *p* < 0.00125), whereas RA only has no significant influence on GERD (OR = 1.04, *p* > 0.0125). In particular, European GERD patients are suffering a 69% increased risk of RA occurrence, which means GERD is a substantial risk factor for RA.

## Data Availability

The original contributions presented in the study are included in the article/Supplementary Material; further inquiries can be directed to the corresponding author.
